# m^6^A-Mediated PPARA Translational Suppression Contributes to Corticosterone-Induced Visceral Fat Deposition in Chickens

**DOI:** 10.3390/ijms232415761

**Published:** 2022-12-12

**Authors:** Zixuan Zhou, Aijia Zhang, Xinyi Liu, Yang Yang, Ruqian Zhao, Yimin Jia

**Affiliations:** 1 Key Laboratory of Animal Physiology & Biochemistry, College of Veterinary Medicine, Nanjing Agricultural University, Nanjing 210095, China; 2 Jiangsu Collaborative Innovation Center of Meat Production and Processing, Quality and Safety Control, Nanjing 210095, China

**Keywords:** adipose tissues, chicken, corticosterone, m^6^A, PPARA

## Abstract

Excess fat deposition in broilers leads to great economic losses and is harmful to consumers’ health. Chronic stress in the life cycle of chickens could be an important trigger. However, the underlying mechanisms are still unclear. In this study, 30-day-old chickens were subcutaneously injected with 2 mg/kg corticosterone (CORT) twice a day for 14 days to simulate long-term stress. It was shown that chronic CORT exposure significantly increased plasma triglyceride concentrations and enlarged the adipocyte sizes in chickens. Meanwhile, chronic CORT administration significantly enlarged the adipocyte sizes, increased the protein contents of FASN and decreased HSL, ATGL, Beclin1 and PPARA protein levels. Moreover, global m^6^A methylations were significantly reduced and accompanied by downregulated METTL3 and YTHDF2 protein expression by CORT treatment. Interestingly, the significant differences of site-specific m^6^A demethylation were observed in exon7 of *PPARA* mRNA. Additionally, a mutation of the m^6^A site in the PPARA gene fused GFP and revealed that demethylated RRACH in PPARA CDS impaired protein translation in vitro. In conclusion, these results indicated that m^6^A-mediated PPARA translational suppression contributes to CORT-induced visceral fat deposition in chickens, which may provide a new target for the treatment of Cushing’s syndrome.

## 1. Introduction

In the poultry industry, excessive fat deposition is considered an undesirable factor, reducing feed conversion and meat quality, causing great economic loss and promising negative effects on consumers’ health [1]. The sustained hypercorticosteronism caused by environmental factors, such as extreme temperature, light intensity, stocking density, toxic gases and feed restriction [2,3], is a potential trigger factor for visceral fat deposition in broiler chickens [4], which is similarly observed in Cushing’s syndrome patients [5]. Extensive studies have reported that glucocorticoids (GC) can affect multiple aspects of adipose tissue biology, including adipogenesis [6,7], lipogenesis and lipolysis [8] and thermogenesis [9,10] in mice and humans. Unlike in mammals, almost 90% de novo fatty acid synthesis occurs in the liver of chickens [11], and the underlying mechanism of GC exposure on adipose tissues are still unclear in chickens.

The nuclear receptor peroxisome proliferator-activated receptor α (PPARA) belongs to the PPARs family, which plays key roles in the regulation of lipid homeostasis and oxidative metabolism [12]. PPARA activation enhances fatty acid oxidation [13], ketogenesis [14], gluconeogenesis [15] and erythroid progenitor self-renewal [16], while global and liver-specific PPARA depletions lead to nonalcoholic fatty liver, diabetes and hypertension [15,17,18]. Recently, Terry et al. [19] reported that adipose-specific PPARA knockout mice have increased lipogenesis in white adipose tissue. Previous studies [20,21] have demonstration that stress and GC induce PPARA expression in cultured hepatocytes and intact livers of mice. However, the mechanism underlying GC-induced PPARA in adipose tissues is poorly understood.

N6-methyladenosine (m^6^A) modifications are the most abundant internal modifications of message RNAs, which influence the stability, splicing and translation of mRNA [22]. M^6^A is demethylated by FTO and methylated by METTL3 and exerts its biological functions through the YTHDF family [23]. Wang et al. [24] and Wu et al. [25] demonstrated that m^6^A is involved in the regulation of adipogenesis through autophagy and controlling the cell cycle. Additionally, Hu et al. [26] reported that corticosterone (CORT) exposure induces hepatocytes lipid accumulation via m^6^A modifications on the 3’UTR of sterol regulatory element-binding transcription factor 1 and stearoyl-CoA desaturase (SCD1) mRNAs.

In this study, we aimed to investigate whether PPARA is involved in CORT-induced adiposity in chickens and to determine whether an m^6^A modification is involved in the post-transcriptional regulation of PPARA.

## 2. Results

### 2.1. Chronic CORT Exposure Induces Visceral Fat Deposition

**Figure 1:**
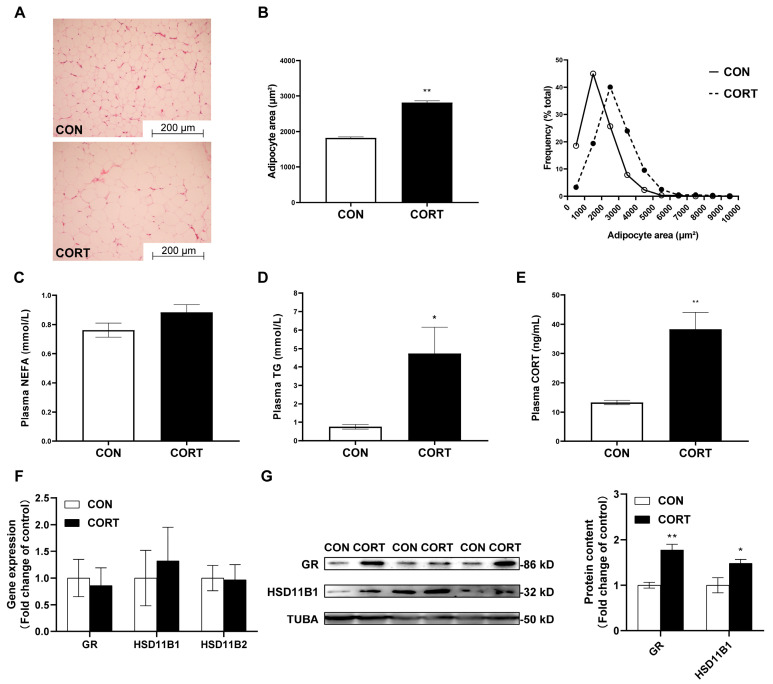
The effects of chronic corticosterone exposure on visceral fat deposition in chickens. (**A**) HE staining for adipose tissue. (**B**) Average area and size frequency distribution in visceral adipocytes. (**C**) Plasma NEFA levels. (**D**) Plasma TG levels. (**E**) Plasma corticosterone levels. (**F**,**G**) The mRNA and protein contents of GR, HSD11B1 and HSD11B2. Values are mean ± SEM of 12 replications in each group. * indicates *p* < 0.05, ** indicates *p* < 0.01.

Chronic CORT exposure significantly increased the average size of adipocytes compared with the CON group (*p* < 0.01, Figure 1A,B). The highest frequency of the visceral fat area was 1500 μm^2^ and 2500 μm^2^ in the CON and CORT group, respectively. In addition, it was observed that the concentrations of plasma TG (*p* < 0.01, Figure 1D) and corticosterone (*p* < 0.05, Figure 1E) were significantly upregulated in the CORT group. Furthermore, excessive CORT exposure significantly promoted the protein contents of GR (*p* < 0.01) and HSD11B1 (*p* < 0.05, Figure 1G) in adipose tissues.

### 2.2. Chronic CORT Exposure Decreases Lipolytic Functions in Visceral Fat Tissue

**Figure 2:**
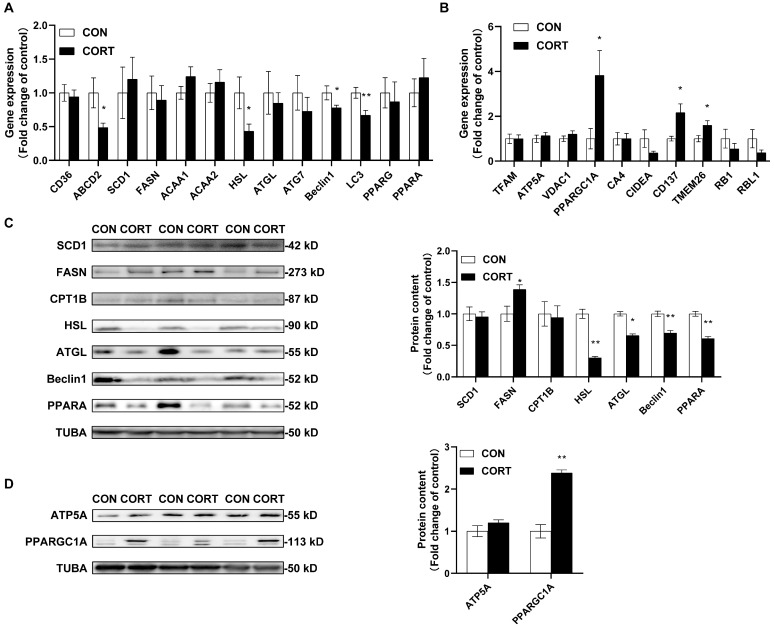
The effects of chronic corticosterone exposure on adipose tissue metabolism in chickens. (**A**) Gene expression for fat acid transportation, synthesis, degradation and adipogenesis in visceral fat tissues. (**B**) Gene expression for mitochondrial functions in visceral fat tissues. (**C**) Protein expression for fat acid transportation, synthesis, degradation and adipogenesis in visceral fat tissues. (**D**) Protein expression for mitochondrial functions in visceral fat tissues. Values are mean ± SEM of 12 replications in each group. * indicates *p* < 0.05, ** indicates *p* < 0.01.

Chronic CORT administration significantly inhibited the gene expression of *ABCD2* (*p* < 0.05), *HSL* (*p* < 0.05), *Beclin1* (*p* < 0.05) and *LC3* (*p* < 0.01) at mRNA levels (Figure 2A), while it significantly enhanced the gene expression of *PPARGC1A* (*p* < 0.01), *CD137* (*p* < 0.05) and *Tmem26* (*p* < 0.05) in the CORT group (Figure 2B). Furthermore, it was observed that the chronic CORT treatment significantly decreased HSL (*p* < 0.01), ATGL (*p* < 0.05), Beclin1 (*p* < 0.01) and PPARA (*p* < 0.01) protein abundances while it significantly increased FASN (*p* < 0.05) and PPARGC1A (*p* < 0.01) protein contents in adipose tissues (Figure 2C,D).

### 2.3. Chronic CORT Exposure Inhibits Global m^6^A Methylation Levels in Visceral Fat Tissue

**Figure 3:**
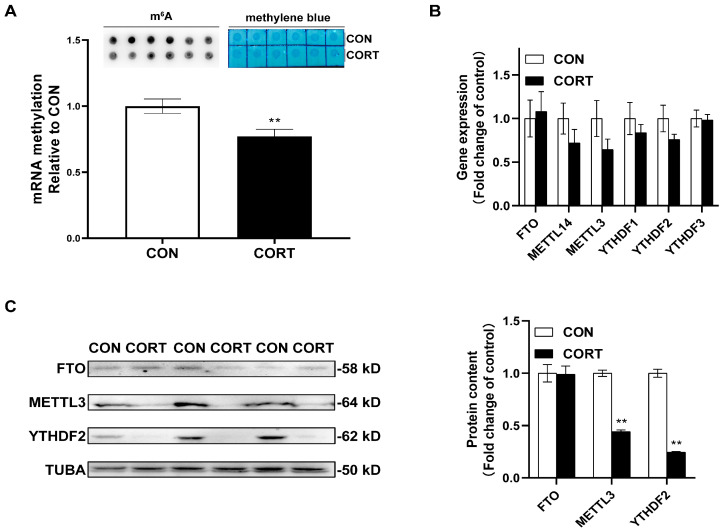
The effects of chronic corticosterone exposure on global m^6^A methylation in visceral fat tissues. (**A**) Dot blot analysis for total RNA in adipose tissues. (**B**) The mRNA contents of m6A methylation related genes. (**C**) The protein contents of m6A methylation-related genes. Values are mean ± SEM of 6 replications in each group. ** indicates *p* < 0.01.

The global m^6^A methylation levels (*p* < 0.01) of the adipose tissues were significantly decreased by chronic CORT administration (Figure 3A). Although chronic CORT exposure had no significant influence on the expression of m^6^A-related genes at mRNA levels (Figure 3B), the protein expression of METTL3 (*p* < 0.01) and YTHDF2 (*p* < 0.01) were significantly decreased by the chronic CORT treatment (Figure 3C).

### 2.4. Chronic CORT Exposure Decreased Site-Specific RNA Methylation in Exon7 of PPARA

**Figure 4:**
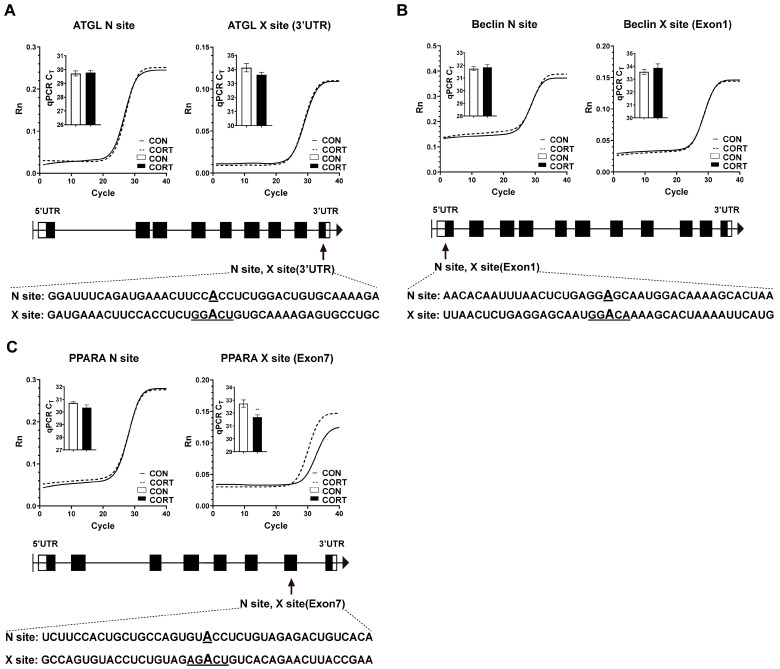
The effects of chronic corticosterone exposure on site-specific RNA methylation in visceral fat tissues. (**A**) C_T_ and amplification curves of potential m^6^A methylation site in 3′UTR of ATGL. (**B**) C_T_ and amplification curves of potential m^6^A methylation site in Exon1 of Beclin1. (**C**) C_T_ and amplification curves of potential m^6^A methylation site in Exon7 CDS of PPARA. Values are mean ± SEM of 12 replications in each group.** indicates *p* < 0.01.

According to our previous m^6^A profiling in the liver of CORT-treated chicken, the potential mRNA m^6^A sites of *ATGL, Beclin1 and PPARA* were predicted by using an online tool SRAMP (http://www.cuilab.cn/sramp, accessed on 8 December 2022). It was shown that there was one potential m^6^A site in the 3′UTR of ATGL (Figure 4A) in Exon1 of Beclin1 (Figure 4B) and in Exon7 of PPARA (Figure 4C). It was observed that chronic CORT exposure did not affect the predicted m^6^A sites in ATGL and Beclin1 but significantly decreased site-specific RNA methylation in the Exon7 of PPARA (*p* < 0.05, Figure 4C).

### 2.5. METTL3 Knockdown Decreased PPARA Protein Content via m^6^A Demethylation in CDS Region

**Figure 5:**
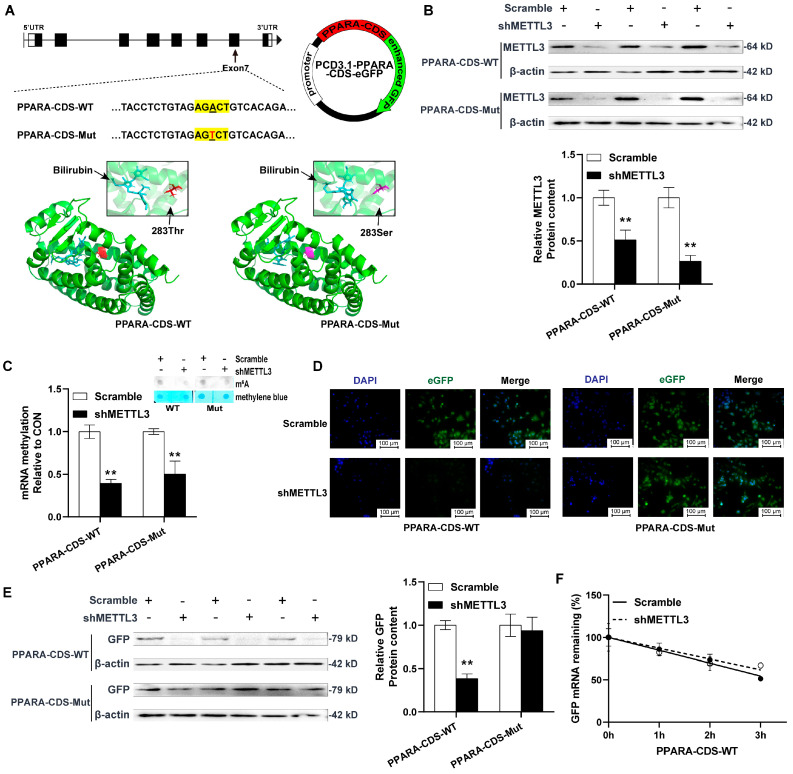
Functional verification of m6A demethylation in CDS of PPARA gene in vitro. (**A**) Schematic representation of mutation in CDS region and molecular visualization of PPARA ligand binding domain. Red: wild-type PPARA (Thr283), purple: mutated PPARA (Ser283), blue: bilirubin. (**B**) The protein levels of METTL3 in PPARA-CDS-WT or PPARA-CDS-Mut. (**C**) Total mRNA m^6^A methylation was measured by DOT-bolt. (**D**) Representative images of 293T cells labelled with GFP (green). Nuclei were counterstained with DAPI (blue), scalebar 100 µm. (**E**) The protein levels of GFP in PPARA-CDS-WT or PPARA-CDS-Mut. (**F**) The half-life of wild-type PPARA-eGFP mRNAs. PPARA-CDS-WT/Mut stands for 293T cells transfected with PPARA-CDS-WT/Mut plasmid. Values are mean ± SEM of 6 replications in each group. ** indicates *p* < 0.01.

To investigate whether m^6^A methylation in the CDS region regulates the expression of PPARA, we generated eGFP-tagged expression constructs encoding a wild-type PPARA CDS containing an AGACT motif with an m^6^A modification (PPARA-CDS-WT), or a mutated CDS containing an AGTCT motif without an m^6^A modification (PPARA-CDS-Mut). Since the A of the m^6^A consensus motif (RRACH) in PPARA is located at the first position of the triple code, a site mutation could cause a change in the sequence of amino acids. Then, we mutated the codon ACT (Thr283) to TCT (Ser283) according to similar amino acid properties. Because the mutated site is located in the ligand binding domain (aa201–aa467) of PPARA, we used AutoDock Tools (Version 1.5.7) to predict whether the mutation could influence the ligands binding. Bilirubin is an agonist of PPARA since it can bind to enhance PPARA activity [27] and alleviate visceral fat deposition [28]. It was observed that the amino acid mutation did not affect the binding of bilirubin as a ligand for PPARA (Figure 5A). Correspondingly, the concentrations of the plasma total bilirubin (CON: 5.85 ± 0.34 vs. CORT: 4.43 ± 0.25, umol/L, the data are present in another reviewed paper) were significantly decreased by CORT exposure in this study.

Additionally, it was observed that shMETTL3 significantly decreased the protein content of METTL3 in HEK293T cells (*p* < 0.01, Figure 5B), as well as the global and PPARA site-specific m^6^A levels (*p* < 0.01, Figure 5C). Interestingly, it was observed that both the GFP fluorescence intensity and protein expression were significantly reduced by METTL3 knockdown only in the PPARA-CDS-WT cells, but not in the CDS-Mut cells (*p* < 0.01, Figure 5D, 5E). Furthermore, Act-D was used to inhibit mRNA transcription in mammalian cells. In this assay, it was observed that the inhibition of m^6^A modification did not affect the half life of PPARA-eGFP mRNAs (Figure 5F).

## 3. Discussion

Cushing’s syndrome is characterized by chronic hypercortisolism. Patients with chronic hypercortisolism show several characteristic clinical features: weight increase, visceral fat deposition and reduced linear growth in children [29]. In our study, chronic CORT exposure significantly decreased the daily weight gain of young chickens (CON: 0.12 ± 0.01 vs. CORT: 0.07 ± 0.01 kg, the data are present in another reviewed paper) but increased the visceral fat weight. Unfortunately, we missed weighing visceral fat. In other ways, the hyperlipidemia and increased average size of the adipocytes could support this evidence. In addition, the lipolytic effects of CORT are concentration dependent [30]. In this study, chickens with chronic hypercortisolism induced by CORT exposure exhibited partial clinical features of Cushing’s syndrome. These results were consistent with previous publications in white- and yellow-feathered broilers [26,31]. Additionally, HSD11B1 is a reductase predominantly expressed in the liver and adipose tissues, which interconverts the inactive glucocorticoid cortisone and its active form cortisol [32]. Here, we observed that chronic CORT exposure exacerbated the protein expression of the GR and HSD11B1 of the adipose tissues in the chickens. Our results indicated that excessive and prolonged CORT is one of the important triggers disrupting lipid metabolism and inducing massive fat deposits in chickens.

In mammals, excessive GC exposure promotes adipogenesis, lipogenesis and brown adipose tissue whitening [33,34,35,36]. Previous publications reported that Beclin1 is a vacuole membrane protein 1 partner in autophagy and lipophagy [37]. Adipocyte-specific Beclin1 −/− mice experienced a severe disruption in autophagy and a dramatically reduced expression of the genes involved in lipid metabolism [38]. Additionally, both ATGL and HSL are the key rate-limiting enzymes involved in the intracellular degradation of TGs [39], and the depletion of ATGL leads to a significant increase in the size of lipid drops and dramatically reduces TAG hydrolysis in vivo and in vitro [40,41]. Furthermore, β-adrenaline stimulated lipolysis in adipose tissue was blunted in HSL−/− mice [42] and were resistant to diet-induced and genetic obesity [43,44]. Additionally, Anthonsen et al. [45] reported that HSLs from chicken adipose tissues are comparable in protein size and activity to HSLs from mammalian species, indicating the existence of HSL proteins in chicken adipose tissues. Therefore, chronic CORT exposure decreased the protein expression of Beclin1, HSL and ATGL, indicating the inhibition of autophagy and lipolysis in the adipose tissues of chickens. Unlike acute GC exposure stimulating lipolysis in vivo [46], long-term GC administration induced adipocyte GR expression [30] and, in our study, suppressed the lipolytic capacity in chicken adipose tissues. Rapidly increasing plasma GC levels provides the basic energy demands to stress adaptation, while prolonged and high GC exposure could induce mitochondrial dysfunction [47,48]. In this study, it seemed that chronic CORT exposure induced mitochondrial biogenesis and browning in adipose tissues. Luijten et al. [49] reported that chronic GC-induced obesity develops independently of UCP1. Recently, Sotome et al. [50] demonstrated that abdominal fat exhibits increased lipolysis probably without increased thermogenesis in white leghorn chickens. Therefore, even though chronic CORT exposure increased browning marker gene expression, white adipose browning could not influence the visceral fat deposition.

PPARA is an important ligand-activated nuclear receptor targeting specific genes involved in lipid oxidation, lipid transport, lipoprotein assembly and ketogenesis [51]. Previous studies have shown that the activation of PPARA reduced adiposity and serum TG in mice with a high-fat diet [52]. Additionally, Carlos et al. [15] reported the hepatic activation of PPARA as a mechanism underlying glucocorticoid-induced insulin resistance in LDL receptor null mice. Moreover, Wake et al. [53] demonstrated that PPARA agonists (fenofibrate) had no effect on cortisol secretion and HSD11B1 expression in human adipose tissue. In this study, decreased plasma bilirubin, an agonist of PPARA, was involved in visceral fat deposition. Our findings were consisted with these publications, which indicated that PPARA inhibition could be a major factor that causes visceral fat deposition by chronic CORT exposure in chickens.

Recent studies have demonstrated that m^6^A methylation plays important roles in regulating adipogenesis and adipose tissue expansion in vivo and in vitro [23,54,55]. Here, our results indicated that CORT-induced visceral fat deposition was associated with m^6^A methylation and occurred in the PPARA exon7. Meanwhile, Miranda et al. [56] reported that PPARA agonists enhance lipid mobilization by targeting the promoter of HSL in 3T3-L1. In this study, Animal-TFDB was used to predict the potential peroxisome proliferator response element in the promoter of chicken HSLs. It was found that there are four putative PPARA binding sites (not shown in the results section), indicating the potential of PPARA in lipolysis by targeting the key enzymes of lipolysis in chickens. Sadly, it was a pity that we did not have enough samples for further analysis.

To date, numerous m^6^A mapping studies have revealed that m^6^A is a selective modification with a transcriptome-wide enrichment in the last exon and 3′ UTR, and that a few are located at the 5′ UTR and within the long internal exon regulating the splicing, expression, decay and translation of RNAs [57,58,59,60]. A previous study demonstrated that m^6^A methylation in the CDS region disrupts tRNA selection and translation elongation dynamics [61]. However, Lin et al. [62] revealed that m^6^A in snail CDS, but not 3′UTR, triggers a polysome-mediated translation of snail mRNA in cancer cells. Moreover, Mao et al. [63] and Wu et al. [64] discovered that m^6^A in mRNA-coding regions promotes translation by resolving mRNA secondary structures and increasing mRNA stability, respectively. The methylation site in the PPARA exon7 is conserved among most of the species (not shown in the results section). Since m6A is a site-specific and context-dependent modification [65], further studies are needed to reveal the role of the m6A site in PPARA exon7 among different species. Here, we found that m6A demethylation in the PPARA exon7 hindered mRNA translation but did not affect mRNA stability. Although our results indicated that decreased YTHDF2 may participate in regulating m^6^A-mediated PPARA translational suppression in adipose tissues, further studies are needed to reveal the delicate mechanisms of m^6^A methylation in PPARA CDS.

## 4. Materials and Methods

### 4.1. Animals and Treatment

Twenty-four one-day-old male Xueshan chickens were purchased from Lihua Animal Husbandry Co., Ltd. (Changzhou City, China) and were raised in the Experimental Animal Center of Nanjing Agricultural University. At 30 days of age, the animals were randomly assigned into control (CON, N = 12) and corticosterone groups (CORT, N = 12). The chickens were subcutaneous injected with normal solvent (15% ethanol) or CORT (2 mg/kg) for 14 days (twice a day, 8:00 A.M and 17:00 PM) [26]. The birds had free access to food and water. At 45 days old, all the chickens were weighted and slaughtered after 12 h of fasting. The abdominal fat samples were rapidly fixed in 4% paraformaldehyde for hematoxylin and eosin (HE) staining, or frozen in liquid nitrogen and then stored at −80 °C for further studies.

All procedures involving laboratory animal use were approved by the Animal Ethics Committee of Nanjing Agricultural University, with the project number 2012CB124703. The slaughter and sampling procedures complied with the “Guidelines on Ethical Treatment of Experimental Animals” (2006) No. 398 set by the Ministry of Science and Technology, China.

### 4.2. HE Staining

Formalin-fixed tissues were embedded with paraffin. After sectioning the tissues, the slides were treated with Xylene followed by ethanol. The cross-sectional area of the adipose tissues was quantified using Image J software.

### 4.3. Plasma Parameters Determination

Commercial biochemical kits were used to determine the concentrations of nonesterified fatty acids (NEFA), cholesterol (CHOL), triglyceride (TG) and bilirubin (BIL) by an automatic biochemical analyzer Hitachi 7020 (Tokyo, Japan) following the manufacturer’s instructions.

### 4.4. RNA Isolation and Real-Time PCR

TRIzol (Tsingke Biotechnology Co., Ltd., Beijing, China) was applied to isolate total RNA by using approximately 200 mg of abdominal fat samples according to the manufacturer’s instructions. One microgram of total RNA was reverse transcribed into cDNA using HiScript II Q RT SuperMix (Vazyme, China). Then, the cDNA was diluted (1:20) for a real-time PCR with Applied Biosystems^®^ QuantStudio™ 6 Flex Real-Time PCR Systems (Thermo Fisher, USA). The primers for qPCR were synthesized by Tsingke Biotechnology (Table A1). PPIA was selected as an internal control to normalize the expression of the target genes. The 2^−ΔΔCt^ method was used to analyze the target genes’ expression.

### 4.5. Western Blot

About 100 mg of adipose tissue samples were homogenized in a RIPA buffer with the protease inhibitor cocktail (Bimake LLC., Houston, TX, USA), and they were centrifuged at 12,000 rpm at 4 °C for 15 min. After removing the upper layer of the lipid, protein concentrations of the supernatants were determined using a Pierce™ BCA Protein Assay Kit (Thermo Scientific, Waltham, MA, USA). Forty micrograms of protein were used for electrophoresis on a 10% SDS-PAGE gel, and they were transferred onto a nitrocellulose membrane and then blocked in 5% skimmed milk at room temperature for 2 h. After that, the immunoblots were incubated at 4 °C overnight with the following primary antibodies: GR (1:1000; Abcam, Cambridge, UK), HSD11B1 (1:1000; Abcam, Cambridge, UK), PPARA (1:1000, Protein Tech, USA), HSL (1:1000; Abcam, Cambridge, UK), ATGL (1:1000; Bioworld, Minnesota, USA), SCD1(1:1000; Abcam, Cambridge, UK), FASN(1:1000; Abcam, Cambridge, UK), CPT1B (1:1000; Bioworld, Minnesota, USA), Beclin1 (1:1000; Bioworld, Minnesota, USA), FTO (1:1000; Abcam, Cambridge, UK), METTL3 (1:2000; Abcam, Cambridge, UK), YTHDF2 (1:2000; Abcam, Cambridge, UK) and GFP (1:2000, Abmart, Shanghai, China). TUBA (1:1000; Bioworld, Minnesota, USA) or β-actin (1:1000; Abcam, Cambridge, UK) was used as the loading control for normalization purposes. Then, the immunoblots were incubated with a secondary antibody (1:; Bioworld, Minnesota, USA) at room temperature for 2 h, and the immunoblots were visualized using enhanced Chemistar™ High-sig ECL Western Blotting Substrate (Tanon, Shanghai, China).

### 4.6. Global and Site-Specific M^6^A Detection

A dot blot assay was used to assess the global m^6^A methylation, as was performed in a previous publication [26]. Additionally, a single-base elongation- and ligation-based qPCR amplification method (SELECT) was performed according to a previous publication with some modifications [66]. In total, 3 µg of RNA were added into 20 µL of a reaction system containing 40 nM PCR adapters (Table A2), 10 µM dNTP and a 2 µL 10 × CutSmart buffer (NEB, Ipswich, USA). The mixtures were annealed in gradient descent from 90 °C to 40 °C by 10 °C per minute. After that, the previous mixtures were added with 0.5 U SplintR ligase (NEB, Ipswich, Burlington, MA, USA), 0.01 U Bst 2.0 DNA polymerase (NEB, Ipswich, USA) and 20 nmol ATP. The final mixtures were incubated at 40 °C for 20 min and denatured at 80 °C for a further 20 min. The cDNA was amplified by real-time PCR to quantify m^6^A at specific sites of target genes. The primers used in the SELECT assay are listed in Table A2.

### 4.7. Plasmids Construction

A full-length CDS of chicken PPARA was cloned into pcDNA3.1-eGFP (Miaolingbio Biotechnology Co., Ltd., Wuhan, China) to generate a pcDNA-PPARA-eGFP plasmid. The linker sequence (Ser-Gly-Gly, AGCGGTGGA) was inserted between PPARA and eGFP. The resulting plasmid was used as a template to generate the demethylase inactive mutant plasmid. A MutUFO™ Fast Mutagenesis Kit (ATG Biotechnology Co., Ltd., Nanjing, China) was used to generate a mutated PPARA-eGFP plasmid through the conversion of AGACT to AGTCT in exon7. Furthermore, shMETTL3-pLKO.1 plasmids (shRNA: 5′-GCCAAGGAACAATCCATTGTT-3′) were synthesized from Nanjing Futuo Biotechnology Co., Ltd., China.

### 4.8. Cell Culture and Transfection

HEK293T cells were cultured in Dulbecco’s Modified Eagle’s Medium with 10% (*v/v*) fetal bovine serum (TRANS, Beijing, China) and maintained in 5% CO_2_ at 37 °C. The cells were seeded into 6-well plates and grown for about 24 h. Then, wild-type (PPARA-CDS-WT) or mutated (PPARA-CDS-Mut) plasmids were transfected together with scramble or shMETTL3, respectively, by using jetPRIME (Polyplus Transfection, Strasbourg, France). After 48 h, the cells were collected and further investigated by qPCR, immunofluorescence and Western blot analysis.

### 4.9. Immunofluorescence Analysis

The cells were fixed with 4% paraformaldehyde in 1×PBS for 30 min at room temperature, washed with PBS three times and then the nuclei were stained with DAPI. The DAPI was excited by the 355 nm laser and its emission collected using a 450/50 filter, while the GFP was excited at 488 nm and detected at 507 nm. The images were captured with a fluorescence microscope (OLYMPUS, Tokyo, Japan).

### 4.10. Deadenylation and Decay Assays

METTL3 shRNA was transfected together with wild-type PPARA-CDS-eGFP plasmids for 48 h. To determine the deadenylated mRNA stability, 10 µg/mL Actinomycin D (Act-D, Aladdin, Xi’an, China) was added to the cells. After incubation at the indicated times (0 h, 1 h, 2 h and 3 h), the cells were collected for total RNA isolation using TRIzol reagent, and then the cDNA was synthesized from an RNA template via a HiScript III 1st Strand cDNA Synthesis Kit (Vazyme, Nanjing, China). The RNA decay rate was determined through real-time PCR according to linear regression. PPIA was used for normalization.

### 4.11. Statistical Analysis

The data were analyzed using GraphPad 8.0 and SPSS 20.0 and were presented as mean ± SEM. The student’s t-test was used to determine the statistical significance. A value *p* < 0.05 was considered statistically significant.

## 5. Conclusions

M^6^A-mediated PPARA translational suppression contributes to CORT-induced visceral fat deposition in chickens. These findings could elaborate deeper insights into the mechanisms of regulating chronic stress-induced fat deposition and provide a new target for the treatment of Cushing’s syndrome.

## Data Availability

Not applicable.

## Abbreviations

CON: control; CORT: corticosterone; CL: Cycloleucine; m^6^A: N6-methyladenosine; TG: triglyceride; NEFA: nonesterified fatty acids; GC: glucocorticoid; DEX: dexamethasone; HE: hematoxylin and eosin; GFP: green fluorescent protein; Act-D: Dactinomycin; GR: nuclear receptor subfamily 3 group C member 1; HSD11B1/2: hydroxysteroid 11-beta dehydrogenase 1/2; TUBA: alpha tubulin; ABCD2: ATP binding cassette subfamily D member 2; ACAA1/2: acetyl-CoA acyltransferase 1/2; ATG7: autophagy-related 7; HSL, hormone sensitive lipase; ATGL, adipose triglyceride lipase; PPARA, peroxisome proliferator activated receptor alpha; PPARG, peroxisome proliferator-activated receptor gamma; FASN, fatty acid synthase; SCD1, stearoyl-CoA desaturase; CD36: CD36 molecule; LC3, microtubule-associated protein 1 light chain 3 beta; TFAM: transcription factor A, mitochondrial; ATP5A: ATP synthase F1 subunit alpha; VDAC: voltage-dependent anion channel 1; PPARGC1A: PPARG coactivator 1 alpha; CA4: carbonic anhydrase 4; CIDEA: cell-death-inducing DFFA-like effector a; CD137: vascular endothelial growth factor A; TMEM26: transmembrane protein 26; RB1: RB transcriptional corepressor 1; RBL1: RB transcriptional corepressor-like 1; FTO, obesity-associated protein; METTL14, methyltransferase-like 14; METTL3/14, methyltransferase-like 3/14; YTHDF1/2/3, YT521-B homology protein families 1/2/3; TUBA; alpha tubulin; β-actin: actin, beta.

## Appendix A

**Table A1:** Primers used in animal and cell experiments.

Target Genes	Primer Sequences (5′ to 3′)	PCR Products (bp)	Amplification Efficiency (%)
GR	TGCACAAGCCCCTGTATCTG	265	102.21%
NM_001037826.1	AATGCTTTCCTCCAGGAGCC		
HSD11B1	TTGGGACTGGTTTTGGCCTT	238	98.84%
XM_025143927.3	TGGCCTTCCTGTGTTTGTGT		
HSD11B2	AGAGAGTGATCGTGACGGGA	294	107.23%
XM_417988.8	CGTCCCCGTTGAAGAAGTCA		
ABCD2	CACACAGACCTTCTCTTTGTACC	136	93.43%
NM_001199395.2	CCTGCAAGTTGGGACTCCAG		
ACAA1	TGGAAAACAGCAAAGCTCGC	117	91.29%
NM_001197288.2	GTTGGGAAGCCAAGGCAAAG		
ACAA2	TAATGGGCATTGGCCCTGTT	280	91.29%
NM_001006571.3	CCAATGCAAGCTGACCCAAC		
ATG7	CTGAGGGGCATGGAGGATGT	100	93.43%
NM_001030592.2	CCCAACTCCCCTTCTGGAAC		
TFAM	AAAGCTTCGCTGCGTGAAAG	242	92.71%
NM_204100.2	CACAGCTCAGGTTACACCGT		
ATP5A	TCTGGCTCCTTACTCAGGCT	143	101.78%
NM_001001935.3	AAAGCAGTCAGAGAGCCACC		
VDAC	TGCTGATCTGGGCAAGTCTG	201	103.54%
NM_001033869.3	TTCTCCGTGAACATCAGCCC		
PPARGC1A	AAAACGGAGGGATGGCGAAT	134	98.03%
NM_001006457.2	TCGAGCAGCTGTGTTCATGT		
CA4	GTGAAGACCCTCGACACTGG	282	100.92%
XM_415893.8	GCTGTTCCTCTTGGACTCCC		
CIDEA	ATGGTTGCAAGCAAAGCTGG	103	104.89%
NM_001195123.2	GTGATGCAAAGCAACAGGCA		
CD137	TTGCCTTGCTGCTCTACCTC	117	93.80%
NM_001025366.3	AGCTGCGCTGATAGACATCC		
TMEM26	TGATGGTTCTGCTCAACGCT	145	91.29%
NM_001199598.2	CCTGTCTCCAGGCAAAGGAG		
RB1	GAAACCTCCACAAACGGCAC	184	99.25%
NM_204419.2	GCCCCAATGCAACACACATT		
RBL1	CACAAAGTGTGAGCCGGTTG	285	94.17%
XM_417312.8	TCCCATGCAGTCTTCGCATT		
HSL	TATGGGAGGTGTCTCGGGTT	231	109.34%
XM_040657096.1	AAGCTCTTCCAGAAGCCCAC		
ATGL	TCACCTTCAGCGTCCAAGTC	267	108.68%
NM_001113291.1	AGCAGGTAGGGGACCATCAT		
PLIN1	GACCACAGCAAGGTACACGA	177	102.56%
XM_015292005.3	GATTGCTGCTGGGAGACCTT		
PPARA	ATGCCAAGGTCTGAGAAGGC	168	108.68%
XM_025150258.2	CCCTGCAAGGATGACTCTGG		
PPARG	ACCTCACGAGGAGTCTTCCA	287	94.92%
NM_001001460.1	GCTTCTCCTTCTCCGCTTGT		
CEBPB	CTCCTACCTGGGCTACCAGT	195	95.30%
NM_205253.2	TTGTACTCGTCGCTGTGCTT		
FASN	GCTAAGATGGCATTGCACGG	300	106.28%
NM_205155.3	TGCCAGAGCCTCCACTATCT		
SCD1	ACACCTTAGGGCTCAATGCC	171	98.84%
NM_204890.1	TGGTGGAGTAGTCGTAGGGG		
CD36	CTCTTTGTGGCCTTTGCACC	291	107.71%
XM_040686380.1	CAGCACAGCACCAATGACAG		
FATP	GGTGAGAAGTGGACCTTCCG	165	102.21%
NM_001039602.2	CGCTTCGATGCTGACTTTCG		
Beclin1	ACAGTGGTCAGTTTGGCACA	285	105.82%
NM_001006332.1	AGAAAGCCACCATCGCATGA		
LC3	GTTTCCAATGCCTGCGTGTT	244	94.54%
NM_001031461.1	AACAGGGTCCTAATGCCAGC		
FTO	TCACCAAGGCGACCTCTACT	88	103.54%
XM_040707054.1	GCTGAACCGAGGTGAAAAGC		
METTL14	ATTCGACCAGGATGGCTGAC	155	99.25%
XM_040698977.1	GACTTGGGTGGTGGTGACTT		
METTL3	ATCCTGGAGCTGCTCAACAC	195	108.20%
XM_040655036.1	AGATTCGTCCGTGTGCTTGT		
YTHDF1	AGTTCAGCATACGGGAGCAG	236	93.07%
XM_040650685.1	CCACCATTACCAGAGAGGGC		
YTHDF2	AAGGCCAAGGCAACAAAGTG	110	94.54%
XM_040690125.1	ATATGCATTGTTCGGCCGGG		
YTHDF3	GTGACCCTCCAATGCCATACT	108	105.82%
NM_001006391.1	GGGTGTTTCCTAATGCCCCA		
PPIA	GTCGTGTTCTTCGACATCGC	265	106.76%
XM_046903390.1	CGAAGAGAAAATGGCGGCG		

**Table A2:** Primers used in SELECT.

Name	X Site	N Site
ATGL-3’UTR-up	tagccagtaccgtagtgcgtgGCAGGCACTCTTTTGCACAG	tagccagtaccgtagtgcgtgTCTTTTGCACAGTCCAGAGG
ATGL-3’UTR-down	CCAGAGGTGGAAGTTTCATCcagaggctgagtcgctgcat	GGAAGTTTCATCTGAAATCCcagaggctgagtcgctgcat
PPARA-Exon7-up	tagccagtaccgtagtgcgtgTTCGGTAAGTTCTGTGACAG	tagccagtaccgtagtgcgtgTGTGACAGTCTCTACAGAGG
PPARA-Exon7-down	CTCTACAGAGGTACACTGGCcagaggctgagtcgctgcat	ACACTGGCAGCAGTGGAAGAcagaggctgagtcgctgcat
PPARA-Lastexon-up	tagccagtaccgtagtgcgtgTTAAAAATCCTTAATACATG	tagccagtaccgtagtgcgtgTGTGACAGTCTCTACAGAGG
PPARA-Lastexon-down	CCCTGTAGATTTCCTGCAGTcagaggctgagtcgctgcat	ACACTGGCAGCAGTGGAAGAcagaggctgagtcgctgcat
BECN1-Exon1-up	tagccagtaccgtagtgcgtgCATGAATTTTAGTGCTTTTG	tagccagtaccgtagtgcgtgTTAGTGCTTTTGTCCATTGC
BECN1-Exon1-down	CCATTGCTCCTCAGAGTTAAcagaggctgagtcgctgcat	CCTCAGAGTTAAATTGTGTTcagaggctgagtcgctgcat
